# Supplementary material for: H128N Substitution in the Sa Antigenic Site of HA1 Causes Antigenic Drift Between Eurasian Avian-like H1N1 and 2009 Pandemic H1N1 Influenza Viruses
Source: Viruses. 2025 Oct 12;17(10):1360. doi: 10.3390/v17101360 (PMC12567644; doi:10.3390/v17101360)
Supplement: Supplementary file 1 [file viruses-17-01360-s001.zip › viruses-3885334-supplementary.pdf]

Table S1. Primers used to generate mutant viruses.

Purpose	Primers (5'-3') [a]	
	Forward	Reverse
TJ312/Mut-1	TTAAAAGCTGATACCCTTTGTATAGGCTACCATGCCAAC	GGCATGGTAGCCTATACAAAGGGTATCAGCTTTTAATGT
TJ312/Mut-2	GACACTGTCGACACAGTATTGGAGAAAAATGTGACTGT	AGTCACATTTTTCTCCAATACTGTGTCGACAGTGTCTGTG
TJ312/Mut-3	ATTTACTGGAAGACAAGCATAATGGGAAACTCTGCAGCC	CAGAGTTTCCCATTATGCTTGTCTTCCAGTAAATTAACTG
TJ312/Mut-4	GAAACTCTGCAAACTGAGAGGAGTGGCCCCCTTACAACTG	TAAGGGGGCCACTCCTCTCAGTTTGCAGAGTTTCCCATT
TJ312/Mut-5	CTCTGCAAACTGAGAGGAGTGGCCCCCTTACAACTGGGAAACTG	CAGTTGTAAGGGGGCCACTCCTCTCAGTTTGCAGAGTTTCCCAT
TJ312/Mut-6	AGATCCCCTTACAACTGGGAAAATGCAACGTAGCAGGATG	ATCCATCCTGCTACGTTGCATTTTCCCAGTTGTAAG
TJ312/Mut-7	TACAACTGGGAAACTGCAACATAGCAGGATGGATCCTTG	TTGCCAAGGATCCATCCTGCTATGTTGCAGTTTCCCAGT
TJ312/Mut-8	CTTGGCAACCCAGAATGTGAATCGCTGTCCACAGCGAGATCGTGGTCTT	GATCTCGCTGTGGACAGCGATTCACATTCTGGGTTGCCAAGGATCCATC
TJ312/Mut-9	TTCGTGGTCTTACATAGTAGAGACTTCAAATTCAAAAAAT	TTTGAATTTGAAGTCTCTACTATGTAAGACCACGAATTCG
TJ312/Mut-10	TAGAGACTTCAAATTCAGACAATGGAGCATGCTACCCCG	GGGTAGCATGCTCCATTGTCTGAATTTGAAGTCTCTAT
TJ312/Mut-11	CAAATTCAAAAAATGGAACATGCTACCCCGGAGAATTTGC	AATTCTCCGGGGTAGCATGTTCCATTTTTTGAATTTGAAG
TJ312/Mut-12	CTACCCCGGAGATTTTATTAATTATGAAGAGTTAAAGGAG	TTAACTCTTCATAATTAATAAAATCTCCGGGGTAGCATGC
TJ312/Mut-13	CTGATTATGAAGAGTTAAGGGAGCAGCTGAGTACAGTTTC	ACTGTACTCAGCTGCTCCCTTAACTCTTCATAATCAGC
TJ312/Mut-14	TAAAGGAGCAGCTGAGTTCAGTTTCTTCATTTGAAAGAT	CTTTCAAATGAAGAAACTGAACTCAGCTGCTCCTTTAACT
TJ312/Mut-15	AAATTTTCCCAAAGACAAGTTCATGGCCACACCATGATAC	TGGTGTGGCCATGAACTTGTCTTTGGGAAAATTTCAAATC
TJ312/Mut-16	AGGCAACTTCATGGCCAAACCATGATACCACCAGAGGTAC	CCTCTGGTGGTATCATGGTTTGGCCATGAAGTTGCCTTTG
TJ312/Mut-17	GGCCACACCATGATTCCGACAAAGGTACCACGGTTTCATG	ACCGTGGTACCTTTGTCGGAATCATGGTGTGGCCATGAAG
TJ312/Mut-18	CACCAGAGGTGTCACGGCTGCATGCCCCCACGCTGGAGCCAACAGCTTT TATCG	TGTTGGCTCCAGCGTGGGGGCATGCAGCCGTGACACCTCTGGTGGTATC ATGGT
TJ312/Mut-19	GCTCCCACTCTGGAGCCAAAAGCTTTTATCGGAATTTACT	AAATTCCGATAAAAGCTTTTGGCTCCAGAGTGGGAGCATG
TJ312/Mut-20	GAGCCAACAGCTTTTATAAGAATTTACTATGGATAGT	ACTATCCATAGTAAATTCTTATAAAAGCTGTTGGCTCCAG
TJ312/Mut-21	TTTATCGGAATTTAATATGGCTAGTAAAGAAAGGAAACTC	CCTTTCTTTACTAGCCATATTAAATTCCGATAAAAGCTGT
TJ312/Mut-22	CTAAGCTCAACCAGACATACATAAACGATAAGGGAAAGGAAGTGCTTGT	TTTCCCTTATCGTTTATGTATGTCTGGTTGAGCTTAGGATAGGAGTTTCT

TJ312/Mut-23	GAAAGGAAGTGCTTGTACTTTGGGGAGTGCACCACCCTCC	GGGTGGTGCACTCCCCAAAGTACAAGCACTTCCTTTCCCT
TJ312/Mut-24	TGCTTGTAATTTGGGGAATTCACCACCCTCCAACTGATAG	TCAGTTGGAGGGTGGTGAATTCCCCAAATTACAAGCACTT
TJ312/Mut-25	CACCCTCCAACTATTGCTGTCCAAGAAAGCCTCTACCAGAATAATCATAC	TTCTGGTAGAGGCTTTCTTGGACAGCAATAGTTGGAGGGTGGTGCACTC
TJ312/Mut-26	CTACCAGAATGCTGATGCATATGTTTCAGTTGGATCATC	ACTGAAACATATGCATCAGCATTCTGGTAGAGGGTTTGTT
TJ312/Mut-27	ATAATCATACATATGTTTTTGTTGGATCATCAAAATACT	TATTTTGATGATCCAACAAAAACATATGTATGATTATTCT
TJ312/Mut-28	TTCAGTTGGAACATCAAGATACTCCAAAAAGTTCAAACCAGAAATAGTAGCAAGACC	TACTATTTCTGGTTTGAACTTTTTGGAGTATCTTGATGTTCCAACTGAAACATATGTAT
TJ312/Mut-29	TCACACCAGAAATAGCAACAAGACCTAAAGTCAGAGAAC	CTGACTTTAGGTCTTGTTGCTATTTCTGGTGTGAACCTTT
TJ312/Mut-30	CTAAAGTCAGAGATCAAGAAGGCAGAATGAATTATTACTG	TTCATTCTGCCTTCTTGATCTCTGACTTTAGGTCTTGCT
TJ312/Mut-31	ACTGGACACTGGTAGAACCAGGGGACACCATAACTTTTG	ATGGTGTCCCCTGGTTCTACCAGTGTCCAGTAATAATTC
TJ312/Mut-32	TGTTAGATCAAGGGGACAAAATAACTTTTGAAGCCACTGG	GTGGCTTCAAAAGTTATTTTGTCCCCTTGATCTAACAGTG
TJ312/Mut-33	GCCACTGGAAATTTAGTAGTACCAAGGTATGCATTTGCATTGAAAAAAGG	TTTCAATGCAAATGCATACCTTGGTACTACTAAATTTCCAGTGGCTTC
TJ312/Mut-34	GCATGCATTTACAATGGAAAGAGATGCTGGATCTGGAATTATGAGGTCGG	TAATTCCAGATCCAGCATCTCTTTCCATTGTAAATGCATGCCATGGTGCT
TJ312/Mut-35	CTAGTTCTGGAATTATCATTTCGGATGCTCAGGTTCAC	ACCTGAGCATCCGAAATGATAATTCCAGAACTAGAACCTT
TJ312/Mut-36	TTATGAGGTCGGATACTCCGGTTCACAATTGCACTAC	GTGCAATTGTGAACCGGAGTATCCGACCTCATAATTCCAG
TJ312/Mut-37	ATGCTCAGGTTCACGATTGCAATACAACGTGCCAAACTCCCCATGGGGC	TGGGGAGTTTGGCACGTTGTATTGCAATCGTGAACCTGAGCATCCGACC
TJ312/Mut-38	CAAAGTGCCAAACTCCCGAGGGGGCCTTGAAAGGCAACCT	TTGCCTTTCAAGGCCCCCTCGGGAGTTTGGCACTTTGTAG
TJ312/Mut-39	CATGGGGCCATAAACACCAGCCTTCCCTTTCAGAATGTAC	AAGGGAAGGCTGGTGTTTATGGCCCCATGGGGAGTTTGGC
TJ312/Mut-40	TTCAGAATGTACATCCCATCACTATTGGGAAATGCC	GGGCATTTCCCAATAGTGATGGGATGTACATTCTGAAAGG
TJ312/Mut-41	AATATGTTAAAAGCACCAAACTGAGAATGGCAACAGGACT	CCTGTTGCCATTCTCAGTTTGGTGCTTTTAACATATTTGG
TJ312/Mut-42	AAAGCACCCAACTGAGACTGGCAACAGGACTAAGAAATAT	TTTCTTAGTCCTGTTGCCAGTCTCAGTTGGGTGCTT
TJ312/Mut-43	CAACAGGACTAAGAAATGTCCCCTCTATTCAATCCAGAGG	CTGGATTGAATAGAGGGGACATTTCTTAGTCCTGTTGCC
GD1536/N128H	TTCATGGCCTCATCATGACTCGGAC	TCCGAGTCATGATGAGGCCATGAAC

[a]The nucleotides highlighted with red color represent the mutated amino acids.
